# Eruption disturbance caused by an odontoma that was partially attached to the crown of the adjacent maxillary first molar, and step lesion excavation for the tooth preservation: A case report

**DOI:** 10.1002/ccr3.2523

**Published:** 2019-11-07

**Authors:** Kenichi Miura, Michiyo Miyashin, Takako Kanao, Atsushi Oishi

**Affiliations:** ^1^ Pediatric Dentistry Clinic for Dentofacial Growth and Development Dental Hospital Tokyo Medical and Dental University (TMDU) Tokyo Japan; ^2^ Department of Pediatric Dentistry Oral Health Sciences Medical and Dental Sciences Graduate School of Medical and Dental Sciences Tokyo Medical and Dental University (TMDU) Tokyo Japan; ^3^ Marine Pediatric Dental Clinic Chigasaki‐city Japan

**Keywords:** complex odontoma, eruption disturbance, eruption guidance, tooth preservation

## Abstract

Despite the partial attachment of a complex odontoma to an impacted tooth, it is possible to guide the tooth to erupt normally and preserve it by step lesion excavation and the prevention of infection.

## Graphical abstract

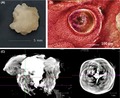

## 1 INTRODUCTION

This report describes a rare case of a complex odontoma that was partially attached to the adjacent impacted maxillary first molar. An impacted tooth that is partially attached to a complex odontoma without the presence of inflammation can be erupted normally by removing the lesion and preventing the infection.

Odontomas often disturb the eruption of the adjacent teeth and are classified into two types: complex and compound odontomas.^1^, ^2^ Complex odontomas contain various types of calcified tissues with no specific tooth structure, whereas compound odontomas exhibit the features of a tooth. The treatment of choice for an odontoma is complete surgical removal of the lesion. This case report describes a patient with an eruption disturbance involving a complex odontoma that was partially attached to the permanent crown of the maxillary first molar. Step lesion excavation was performed to preserve the permanent molar.

## 2 CASE REPORT

**Figure 1:**
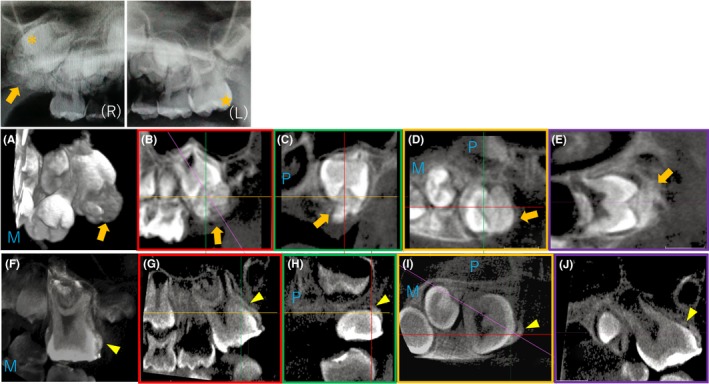
Panoramic radiograph (R, Right; L, Left) and CBCT images at 6 y and 8 mo of age (A‐E) and 8 y and 2 mo of age (F‐J). A radiopaque semicircular lesion (arrow) covered the occlusal surface of the impacted #16. Root formation was delayed when compared with #26 (★) (P, Palatal; M, Mesial)

**Figure 2:**
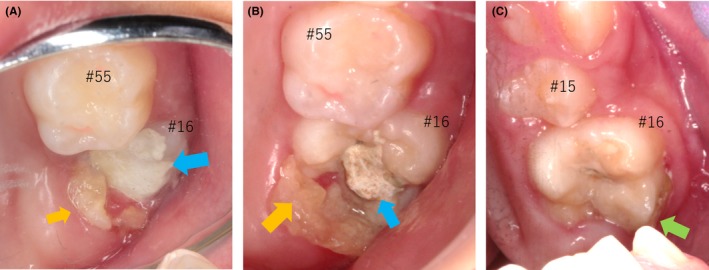
The eruption series of #16 during the treatment period (mirror image). At 7 y and 8 mo of age (12 mo after the start of the treatment), the chalky‐white occlusal surface of the molar was filled with carboxylate cement (blue arrow). The hard tissue of the lesion (yellow arrow) was located adjacent to the tooth (A). At 8 y and 1 mo (17 mo after starting the treatment), the remaining lesion was removed under local anesthesia (yellow; B). At 9 y and 4 mo of age (32 mo after starting the treatment), the adhesive resin was applied to the crown of the molar and the remnants of the lesion to prevent further infection (green arrow; C).

A male patient aged 6 years and 8 months was referred to our pediatric dental clinic for hard‐tissue removal around the crown of an impacted maxillary right first molar. The medical histories of the patient and his family were normal. Panoramic radiography and cone‐beam computed tomography (CBCT) revealed that less than one‐fourth of the root was formed, and it was shorter than that of the corresponding left maxillary molar; additionally, the crown was covered by a radiopaque lesion (Figure 1). The differential diagnosis included odontoma, retention of the right maxillary first molar, and delayed root formation. The treatment plan involved the removal of the lesion to facilitate the eruption of the molar. During the first surgical procedure, which was performed under local anesthesia, the hard tissue covering the tooth was exposed to the oral cavity; however, the lesion could not be separated from the tooth. In due course, the tooth gradually emerged along with the lesion attached to it. A step lesion excavation was planned using an ultrasonic bone scalpel (Varios G95 and 970; Nakanishi Inc, Tochigi, Japan) and diamond bur. The procedure was painless and repeated five times over a period of 9 months from the age of 7 years and 3 months. At 7 years and 8 months of age, the occlusal surface of the molar located beneath the gingiva demonstrated a chalky‐white appearance and was filled with carboxylate cement to prevent pulp infection. Instructions on the use of a tuft brush were given to the patient's parents (Figure 2).

At 8 years and 2 months of age, CBCT revealed remnants of the lesion, which appeared to be attached to the pulp (Figure 1). Therefore, the stepwise removal of the lesion was stopped, and an adhesive resin (Bondfill SB; Sun Medical Company, Ltd., Shiga, Japan) was applied to both the crown and the lesion as a protective measure (Figure 2). At 9 years of age, the molar demonstrated occlusal contact with the opposing mandibular first molar, a positive reaction to the electrical pulp test, and normal tooth mobility.

**Figure 3:**
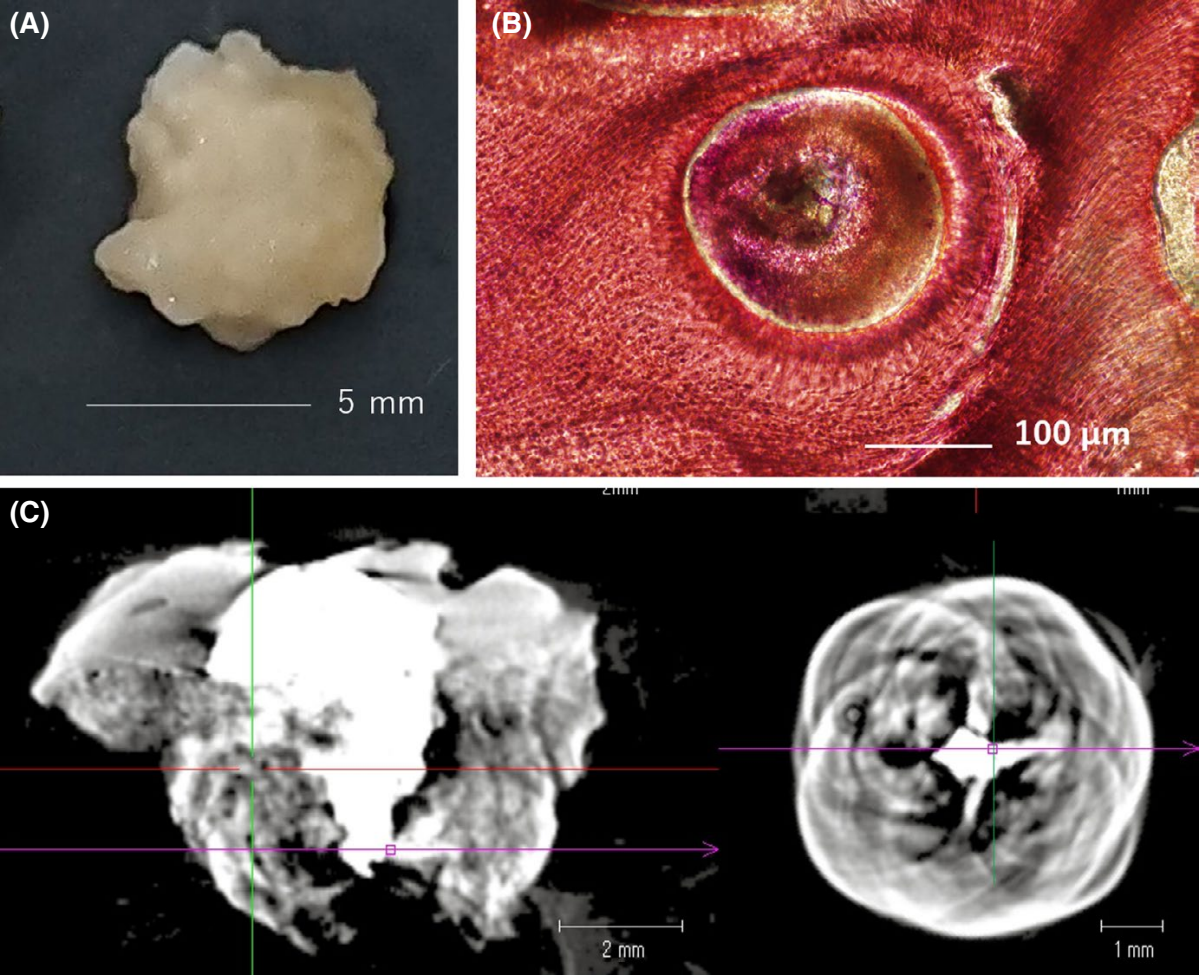
Inspection of the lesion fragment. The diameter of the removed fragment was approximately 5 mm. The surface was uneven, hard, and shiny like that of enamel (A). Hematoxylin‐ and eosin‐stained sections showed concentric cross‐striation, and the diameter of the ring in the center was approximately 200 μm (B). MicroCT sections depicted a microporous organization where the size of the hole was about 200 μm, and the diameter of the X‐ray impermeable area at the center was not >2 mm (C).

The removed lesion fragments had enamel‐like, glossy, and irregular surfaces. Histopathology conducted using hematoxylin and eosin staining revealed the presence of multiple holes accompanied by concentric cross‐striation rings, while microcomputed tomography (microCT, SMX‐100CT; Shimadzu Corporation, Kyoto, Japan) showed a highly calcified lesion with an X‐ray impermeable area at the center (Figure 3). Accordingly, the lesion was diagnosed as an enamel‐like tissue‐dominant complex odontoma that was partially attached to the first molar.

## 3 DISCUSSION

The cross‐striations observed in the sections obtained from the lesion were similar to that normally seen in the enamel. Moreover, X‐ray impermeability in the microCT images indicated the presence of areas with enamel‐like hardness. During the initial operation, the removal of the lesion was abandoned as it could not be separated from the molar. Due to the nature of this lesion, utilization of an ultrasonic bone scalpel and diamond bur in a step excavation would be reasonable.

Previous studies have reported that treatment^1^, ^3^ of odontoma involving the complete removal of the lesion and the tooth^3^, ^4^, ^5^ is generally required when it is seen to be impacting on either tooth eruption or the tooth itself. To the best of our knowledge, this is the first report of a complex odontoma that was partially attached to the crown of the adjacent molar and was gradually removed in order to preserve the tooth. We believe that the treatment plan involving step lesion excavation, tooth surface management, and the use of the CBCT findings was justified in this case. However, as the tooth mechanical and adhesive properties are still unclear, subsequent restoration should be considered carefully to ensure longer tooth prognosis.

## 4 CONCLUSION

An impacted tooth that is partially attached to a complex odontoma without the presence of inflammation can be erupted normally by removing the lesion and preventing the occurrence of infection.

## CONFLICT OF INTEREST

The authors declare no conflicts of interests.

## AUTHOR CONTRIBUTIONS

MM: mainly performed the dental treatment. TK: contributed the oral hygiene management during the treatment and observation period. AO and KM: examined the radiographic and pathological findings, and previous study. KM, MM, TK, and AO: drafted and reviewed the manuscript, and all approved the submission.
